# Fragments of peer review: A quantitative analysis of the literature (1969-2015)

**DOI:** 10.1371/journal.pone.0193148

**Published:** 2018-02-21

**Authors:** Francisco Grimaldo, Ana Marušić, Flaminio Squazzoni

**Affiliations:** 1 Department of Computer Science, University of Valencia, Burjassot, Valencian Community, Spain; 2 Department of Research in Biomedicine and Health, University of Split, Split, Split-Dalmatia, Croatia; 3 Department of Economics and Management, University of Brescia, Brescia, Lombardy, Italy; Max Planck Society, GERMANY

## Abstract

This paper examines research on peer review between 1969 and 2015 by looking at records indexed from the Scopus database. Although it is often argued that peer review has been poorly investigated, we found that the number of publications in this field doubled from 2005. A half of this work was indexed as research articles, a third as editorial notes and literature reviews and the rest were book chapters or letters. We identified the most prolific and influential scholars, the most cited publications and the most important journals in the field. Co-authorship network analysis showed that research on peer review is fragmented, with the largest group of co-authors including only 2.1% of the whole community. Co-citation network analysis indicated a fragmented structure also in terms of knowledge. This shows that despite its central role in research, peer review has been examined only through small-scale research projects. Our findings would suggest that there is need to encourage collaboration and knowledge sharing across different research communities.

## Introduction

Peer review is central to research. It is essential to ensure the quality of scientific publications, but also to help the scientific community self-regulate its reputation and resource allocation [1]. Whether directly or indirectly, it also influences funding and publication [2]. The transition of publishing and reading to the digital era has not changed the value of peer review, although it has stimulated the call for new models and more reliable standards [3–5].

Under the impact of recent scandals, where manipulated research passed the screening of peer review and was eventually published in influential journals, many analysts have suggested that more research is needed on this delicate subject [6–10]. The lack of data and robust evidence on the quality of the process has led many observers even to question the real value of peer review and to contemplate alternatives [11–13].

This study aims to provide a comprehensive analysis of peer review literature from 1969 to 2015, by looking at articles indexed in Scopus. This analysis can help to reveal the structure of the field by finding the more prolific and influential authors, the most authoritative journals and the most active research institutions. By looking at co-authorship and co-citation networks, we measured structural characteristics of the scientific community, including collaboration and knowledge sharing. This was to understand whether, despite the growing number of publications on peer review in the last years, research is excessively fragmented to give rise to a coherent and connected field.

Finally, it is important to note that the period covered by our analysis is highly representative. Indeed, although many analysts suggested that peer review is deeply rooted in the historical evolution of modern science since the 17^th^ century [14], recent historical analysis suggested that as an institutionalized system of evaluation in scholarly journals was established systematically only about 70 years ago, where also the terms “peer review” and “referee” became common currency [15].

## Results

**Fig 1:**
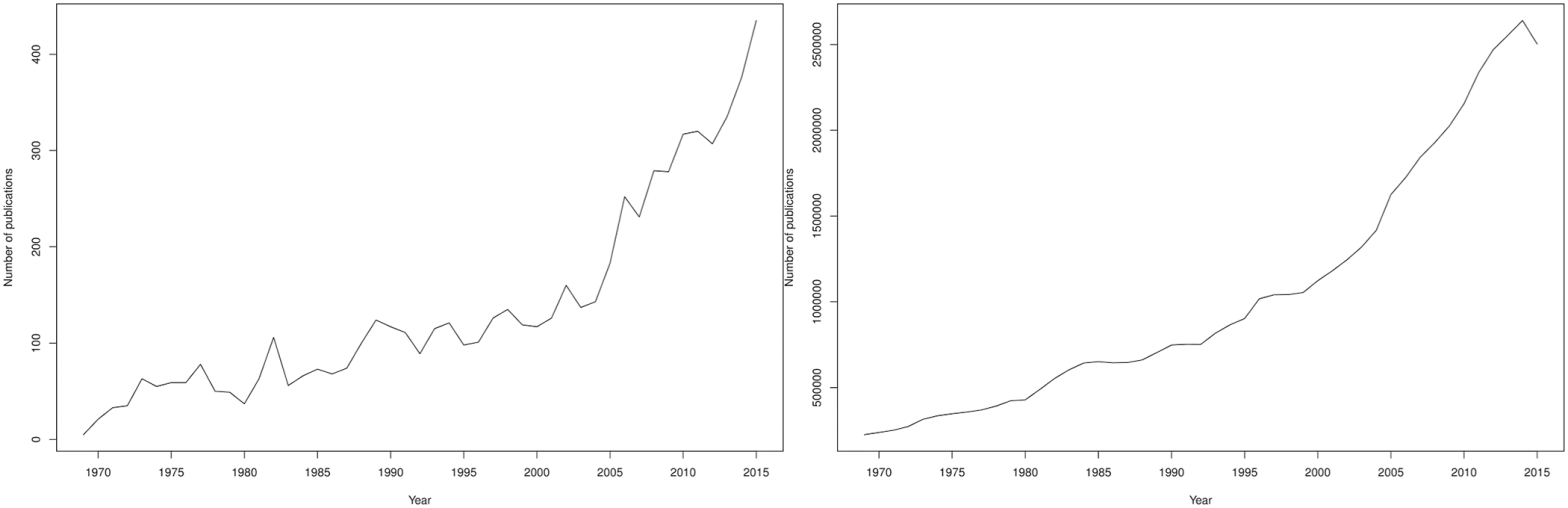
Number of publications about peer review. Number of records on peer review (left) and number of records published in English on any topic from 1969 to 2015 in Scopus (right).

Fig 1 (left panel) shows that the number of publications on peer review doubled from 2005. From 2004 to 2015, the annual growth of publications on peer review was 12% on average, reaching 28% and 38% between 2004–2005 and 2005–2006, respectively. The volume of published research grew more than the total number of publications, which had an average growth of 5% from 2004 to 2015, to reach 15% in 2004–2005 (Fig 1, right panel). The observed peak-based dynamics of this growth can be related to the impact of the International Congresses on Peer Review and Biomedical Publication, which were regularly held every four years starting from 1989 with the *JAMA* publishing abstracts and articles from the second, third and fourth editions of the congress [10]. This was also confirmed by data from PubMed and Web of Science (see Figure A in S1 Appendix).

**Fig 2:**
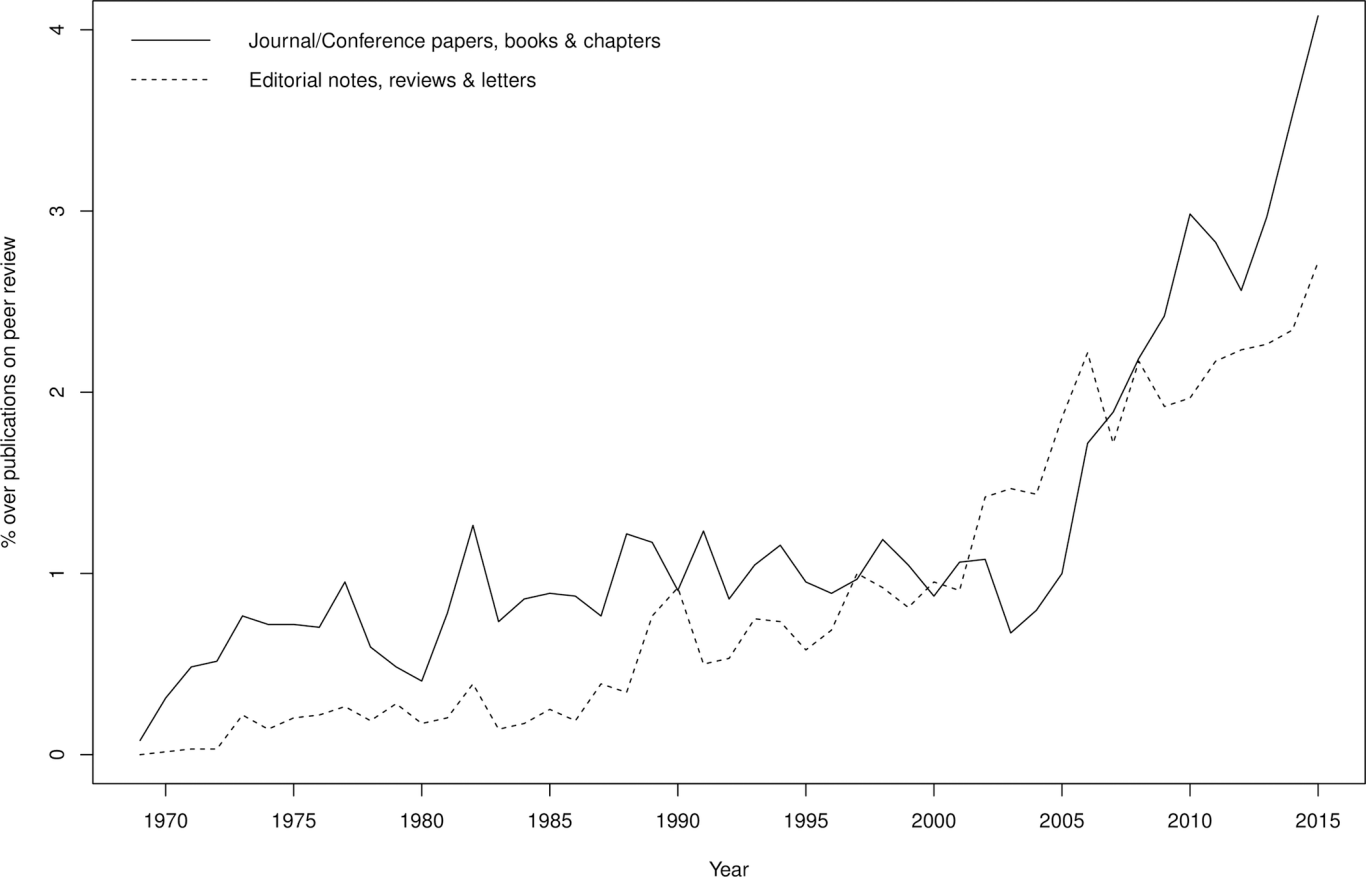
Percentage of records on peer review per type and year (Scopus data).

About half of the records were journal articles, the rest mostly being editorial notes, commentaries, letters and literature reviews (see Figure B in S1 Appendix). However, the number of original research contributions, e.g., research articles, book chapters or conference proceedings papers, increased from 2005 onward to exceed the number of editorial notes, reviews and letters (Fig 2). This would indicate that empirical, data-driven research is increasing recently.

**Fig 3:**
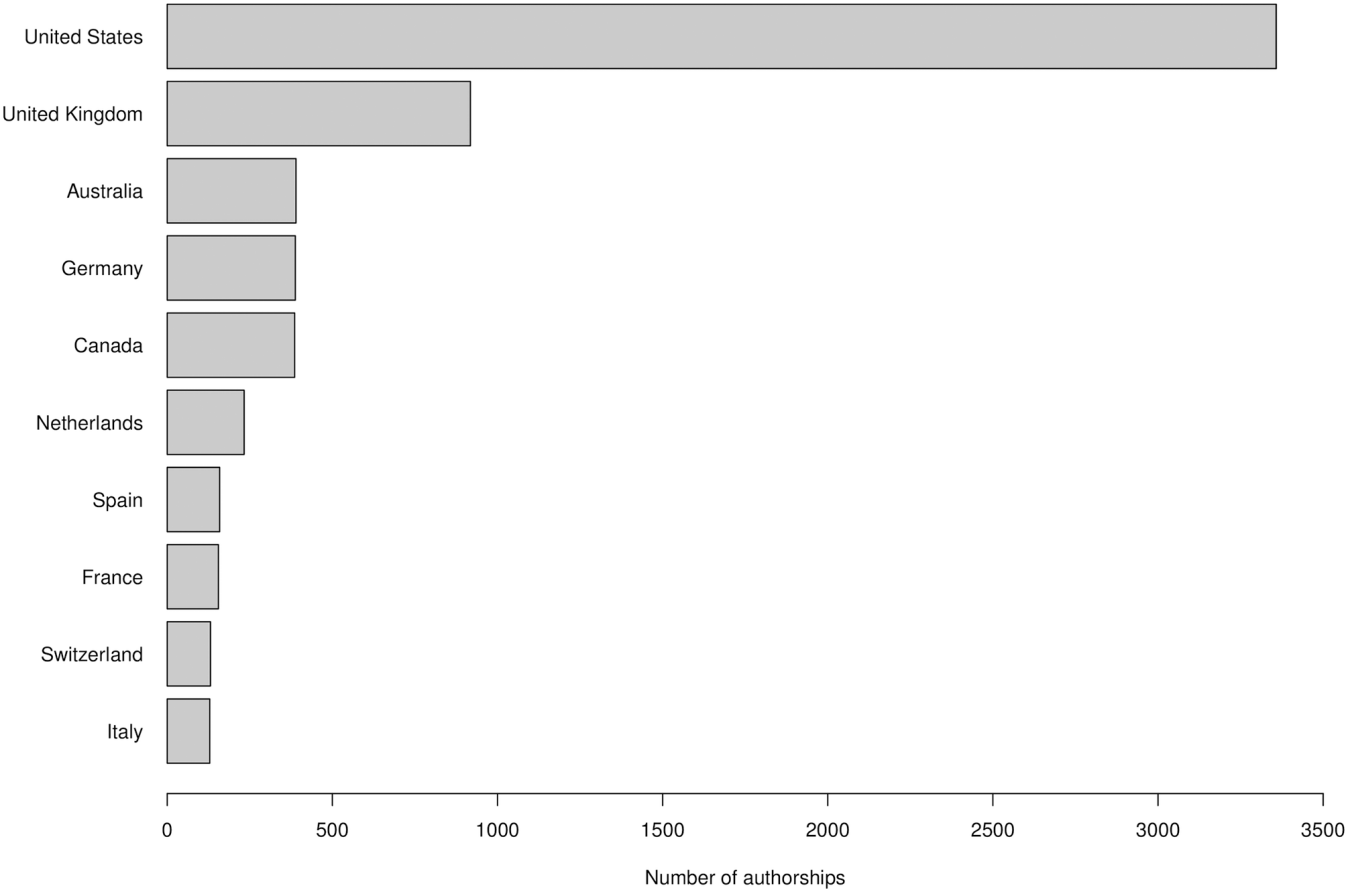
The top 10 countries in which research on peer review is performed (Scopus data).

**Fig 4:**
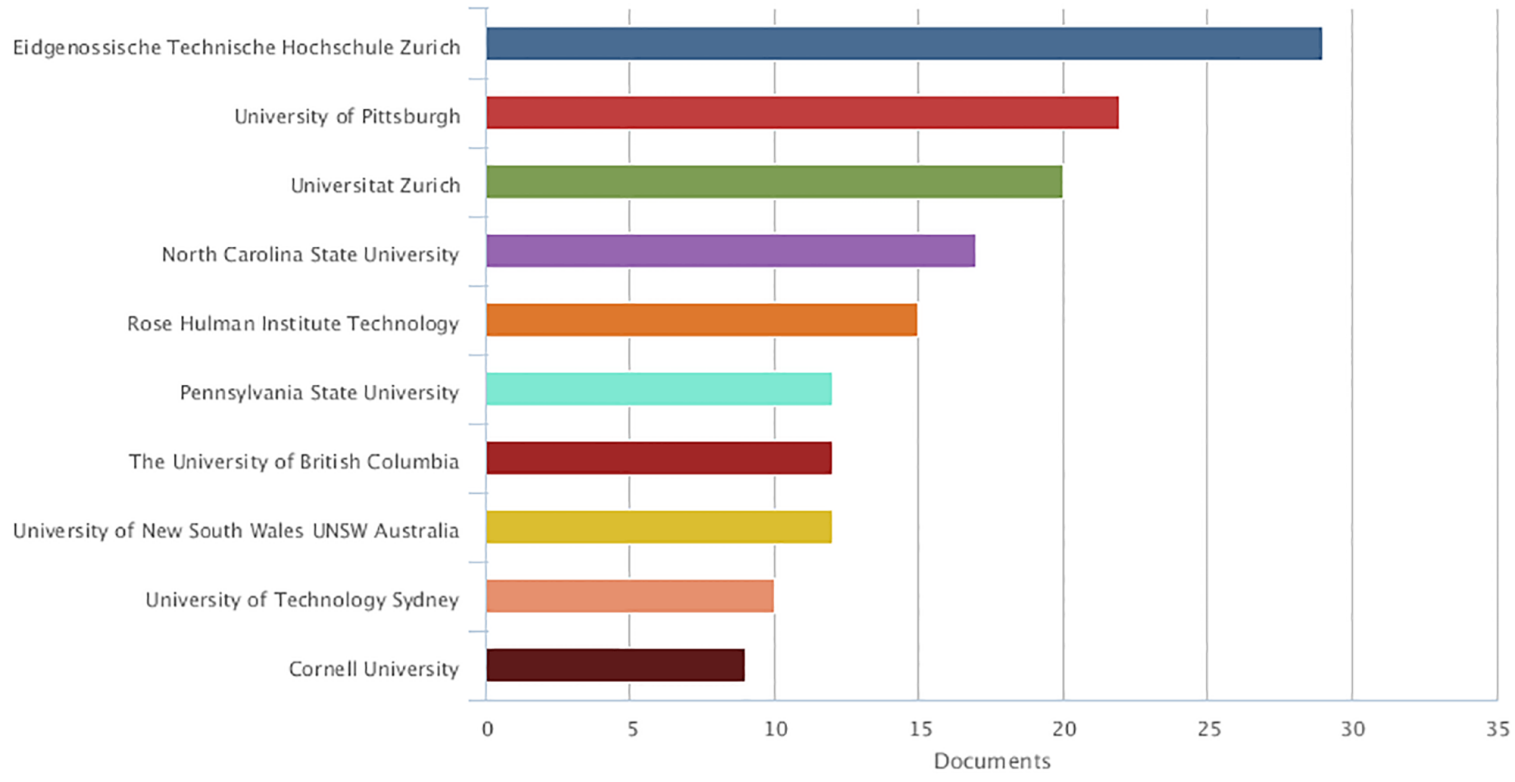
The top 10 institutions in which research on peer review is performed (Scopus data).

Fig 3 shows the top 10 most productive countries by origin of research authors. Peer review is studied predominantly in the US, followed by the UK, Australia, Canada and Germany. While this may also be due to the size-effect of these communities, previous studies have suggested that peer review is intrinsic especially to the Anglo-Saxon institutional context [16]. However, if we look at the top 10 most productive institutions, in which it is probable that research has been cumulative and more systematic, we also found two prominent European institutions, the ETH Zurich and the University of Zurich (Fig 4). This indicates that research on peer review is truly international.

**Fig 5:**
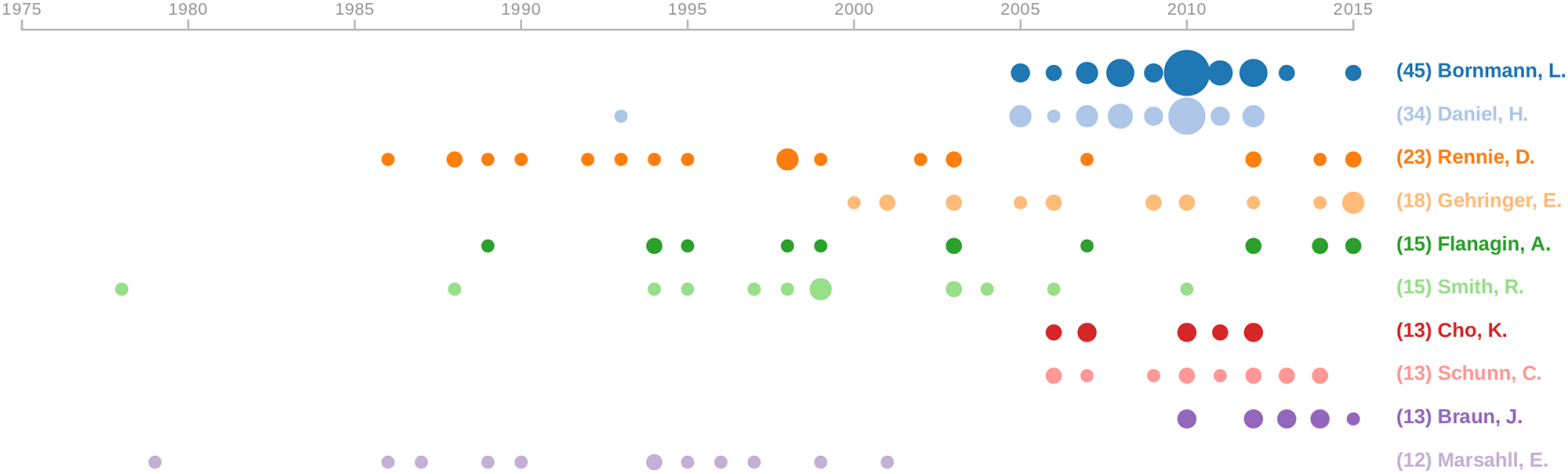
Number of publications per year for the top 10 most prolific authors in sample 1 (Scopus data).

Fig 5 shows the most prolific authors. While the top ones are European social scientists, i.e., Lutz Bornmann and Hans-Dieter Daniel, who published 45 and 34 papers, respectively, the pioneers of research on peer review were medical scholars and journal editors, such as Drummond Rennie, Richard Smith and Annette Flanagin.

**Fig 6:**
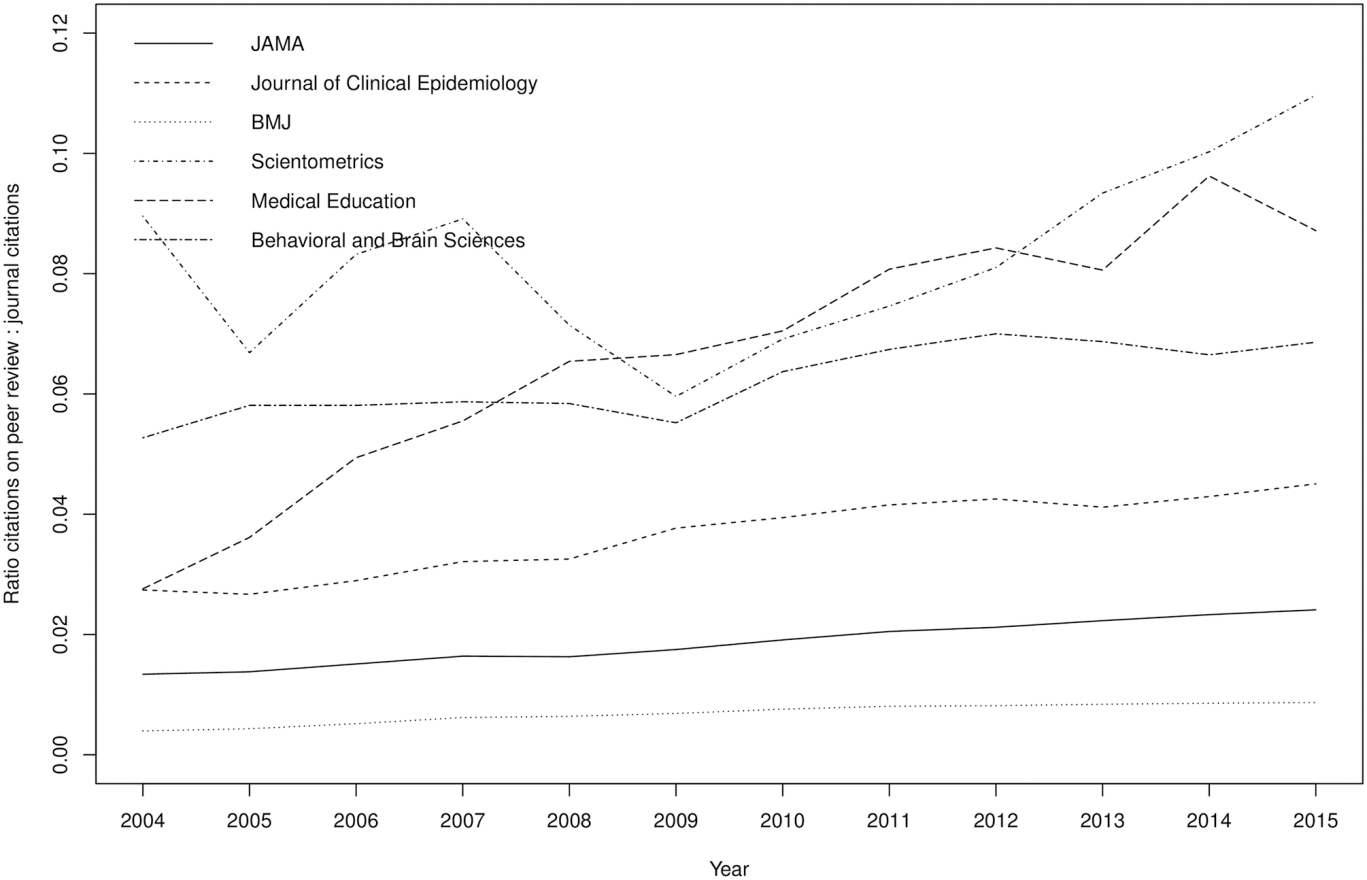
Ratio between the number of citations of papers on peer review and the total number of citations by the journal on sample 1 (Scopus data).

Among publication outlets, the journals that published on peer review most frequently were: *Science* (n = 136 papers), *Nature* (n = 110), *JAMA* (n = 99), *Scientometrics* (n = 65), *Behavioral and Brain Sciences* (n = 48), *Chemical and Engineering News* (34), *Academic Medicine* (32), *Australian Clinical Review* (32), *Learned Publishing* (n = 31) and *Research Evaluation* (n = 31). However, research articles on peer review were published mostly by *JAMA* (n = 62), *Behavioral and Brain Sciences* (n = 44) and *Scientometrics* (n = 42). This means that top journals such as *Science* and *Nature* typically published commentaries, editorial notes or short surveys, while research contributions have mostly been published elsewhere. If we look at the impact of research on peer review on journal citations (see Table A in S1 Appendix), the impact has been minimal with the exception of *Scientometrics*, whose articles on peer review significantly contributed to the journal’s success (10.97% of the whole journal citations were received by articles on peer review). However, it is worth noting that the contribution of research on peer review in terms of journal citations has been increasing over time (see Fig 6 for a restricted sample of journals listed in Table B in S1 Appendix).

Among the most important topics, looking at the keywords revealed that research has preferably examined the connection between peer review and “quality assurance” (103 papers), “publishing” (93), “research” (76), “open access” (56), “quality improvement” (47), “evaluation” (46), “publication” (44), “assessment” (41), “ethics” (40) and “bibliometrics” (39). The primacy of the link between peer review and the concept of “quality” was confirmed by looking at nouns/verbs/adjectives in the paper titles (“quality” appearing 527 times against “journal” appearing 454 times or “research”, 434 times) and in the abstracts (“quality” recurring 2208 times against “research” 2038 or medical 1014 times). This would confirm that peer review has been mainly viewed as a “quality control” process rather than a collaborative process that would aim at increasing the knowledge value of a manuscript [17].

Data showed that research on peer review is typically pursued in small collaborative networks (75% of the records had less than three co-authors), with the exception of one article published in 2012, which was co-authored by 198 authors and so was excluded by the following analysis on co-authorship networks to avoid statistical bias (see Figure D in S1 Appendix). Around 83% of the co-authorship networks included less than six authors (see Figure E in S1 Appendix). The most prolific authors were also those with more co-authors, although not those with a higher average number of co-authors per paper (see Table E in S1 Appendix).

The most prolific authors from our analysis were not always those instrumental in connecting the community studying peer review (e.g., compare Table 1 and Fig 5). Fragmentation and small-scale collaboration networks were dominant (e.g., see Table B and Figure E in S1 Appendix). We found 1912 clusters with an average size of 4.1, which is very small. However, it is important to emphasize certain differences in the position of scientists in these three samples. When excluding records published in medicine journals, we found a more connected co-authorship network with scientists working in more cohesive and stable groups, indicated by the lower number of clusters, higher density and shorter diameter in sample 3 in Table 2, which is not linearly related to decreasing numbers of nodes and edges.

**Table 1:** The top 10 most central authors in the co-authorship networks (Scopus data).

Sample 1		Sample 2		Sample 3	
Author	Betweenness	Author	Betweenness	Author	Betweenness
Bosch, F.	6885	Bosch, F.	2842	Schunn, C.D.	185
Cobo, E.	6388	Cobo, E.	2590	Cho, K.	157
Altman, D.G.	5751	Flanagin, A.	2319	Gehringer, E.F.	126
Flanagin, A.	5326	Altman, D.G.	1870	Lee, R.	108
Rennie, D.	3021	Godlee, F.	1335	Allen, L.	102
Marusic, A.	2956	Rennie, D.	892	Jones, L.S.	90
Moher, D.	1883	Cook, J.	792	Terveen, L.	72
Justice, A.D.	1705	Marusic, A.	750	Bollen, J.	62
Godlee, F.	1414	Johnson, N.	693	Van Leeuwen, T.N.	59
Cook, J.	1264	Boutron, I.	606	Trevisan, M.	56

**Table 2:** Network statistics of the co-authorship networks in the three samples (Scopus data).

Sample 1		Sample 2		Sample 3	
Metric	Value	Metric	Value	Metric	Value
N° Nodes	7971	N° Nodes	5590	N° Nodes	2753
N° Edges	18689	N° Edges	12657	N° Edges	5309
Density	0.0006	Density	0.0008	Density	0.001
Diameter	14	Diameter	12	Diameter	8
N° Clusters	1910	N° Clusters	1378	N° Clusters	790
Cluster size (avg)	4.1	Cluster size (avg)	4.1	Cluster size (avg)	3.5
Cluster size (std)	5.8	Cluster size (std)	4.6	Cluster size (std)	3.0

To look at this in more detail, we plot the co-authorship network linking all authors of the papers on peer review. Each network’s node was a different author and links were established between two authors whenever they co-authored a paper. The greater the number of papers co-authored between two authors, the higher the thickness of the corresponding link.

**Fig 7:**
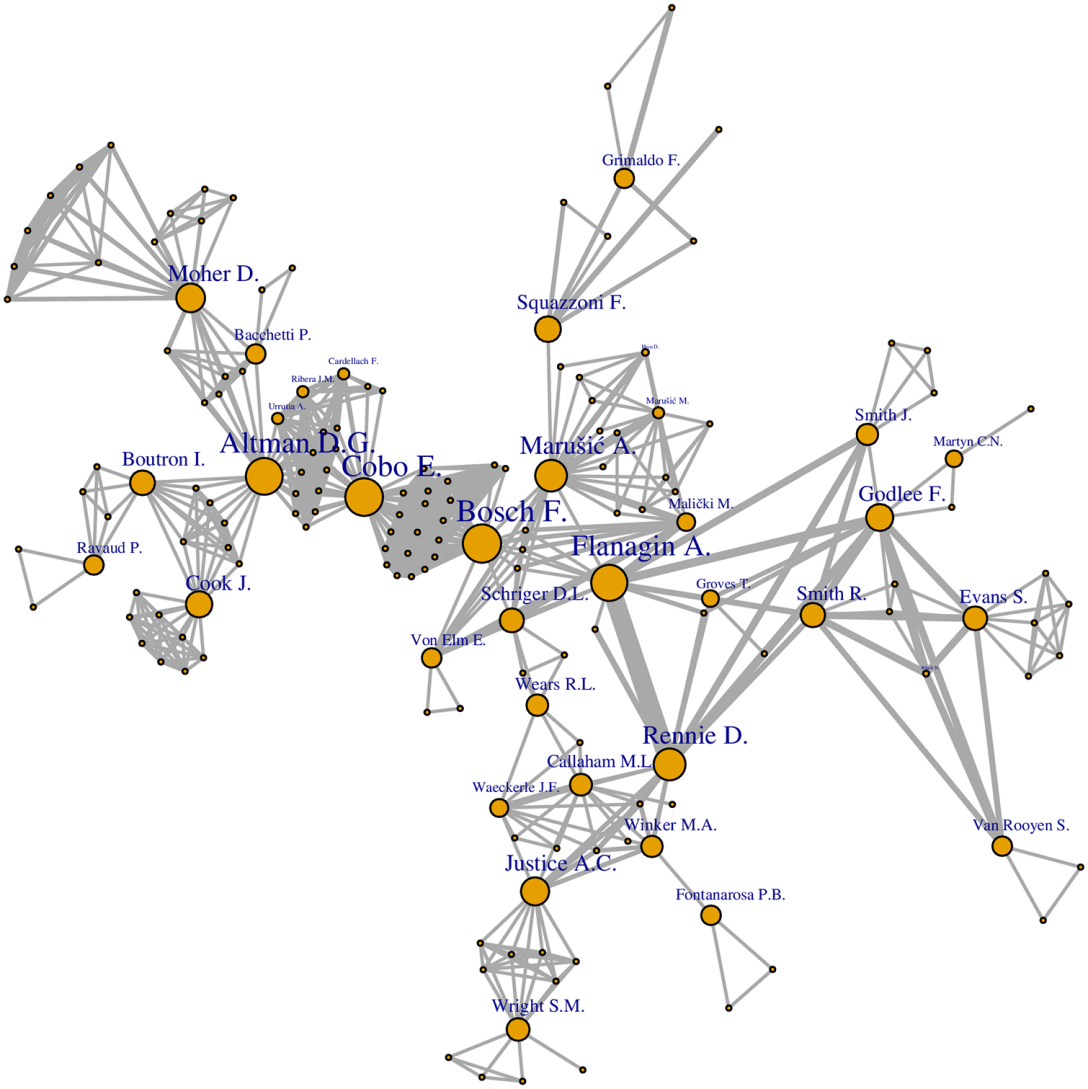
Internal connections in the biggest community of scientists working on peer review (sample 1). Note that the node size refers to the author’s betweeness centrality.

**Fig 8:**
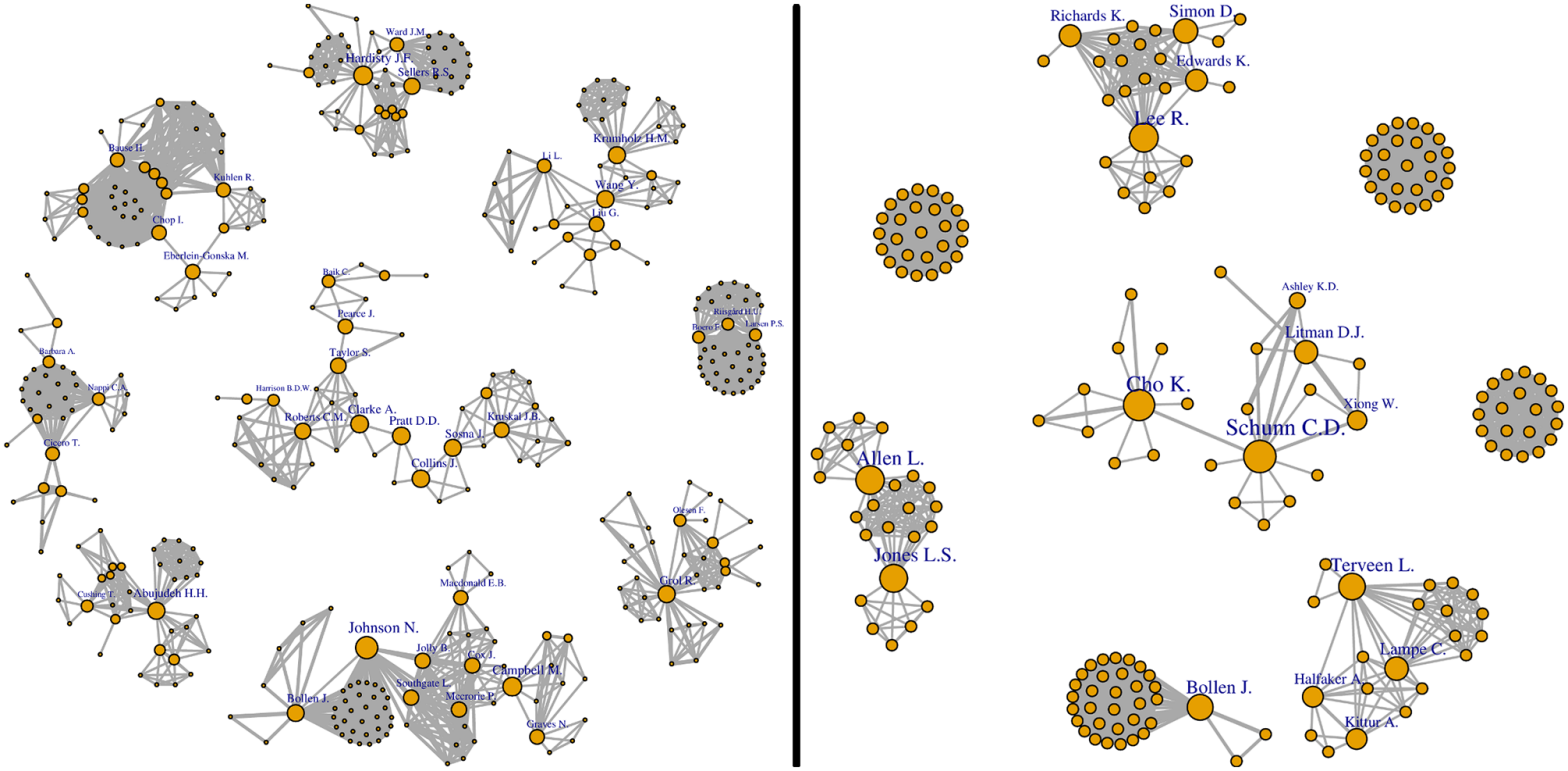
Biggest communities of scientists working on peer review. Sample 1 on the left, i.e., sample 3 on the right, i.e., outside medicine). Note that the node size refers to the author’s betweeness centrality.

The co-authorship network was highly disaggregated, with 7971 authors forming 1910 communities (Table 1). With the exception of a large community of 167 researchers and a dozen of communities including around 30 to 50 scientists, 98% of communities had fewer than 15 scientists. Note that the giant component (n = 167 scientists) represents only 2.1% of the total number of scientists in the sample. It included co-authorship relations between the top 10 most central authors and their collaborators (Fig 7). The situation is different if we look at various largest communities and restrict our analysis to research articles and articles published in non-medicine journals (Fig 8). In this case, collaboration groups were more cohesive (see Fig 8, right panel).

In order to look at the internal structure of the field, we built a co-citation network that measured relations between cited publications. It is important to note that here a co-citation meant that two records were cited in the same document. For the sake of clarity, we reported data only on cases in which co-citations were higher than 1.

**Fig 9:**
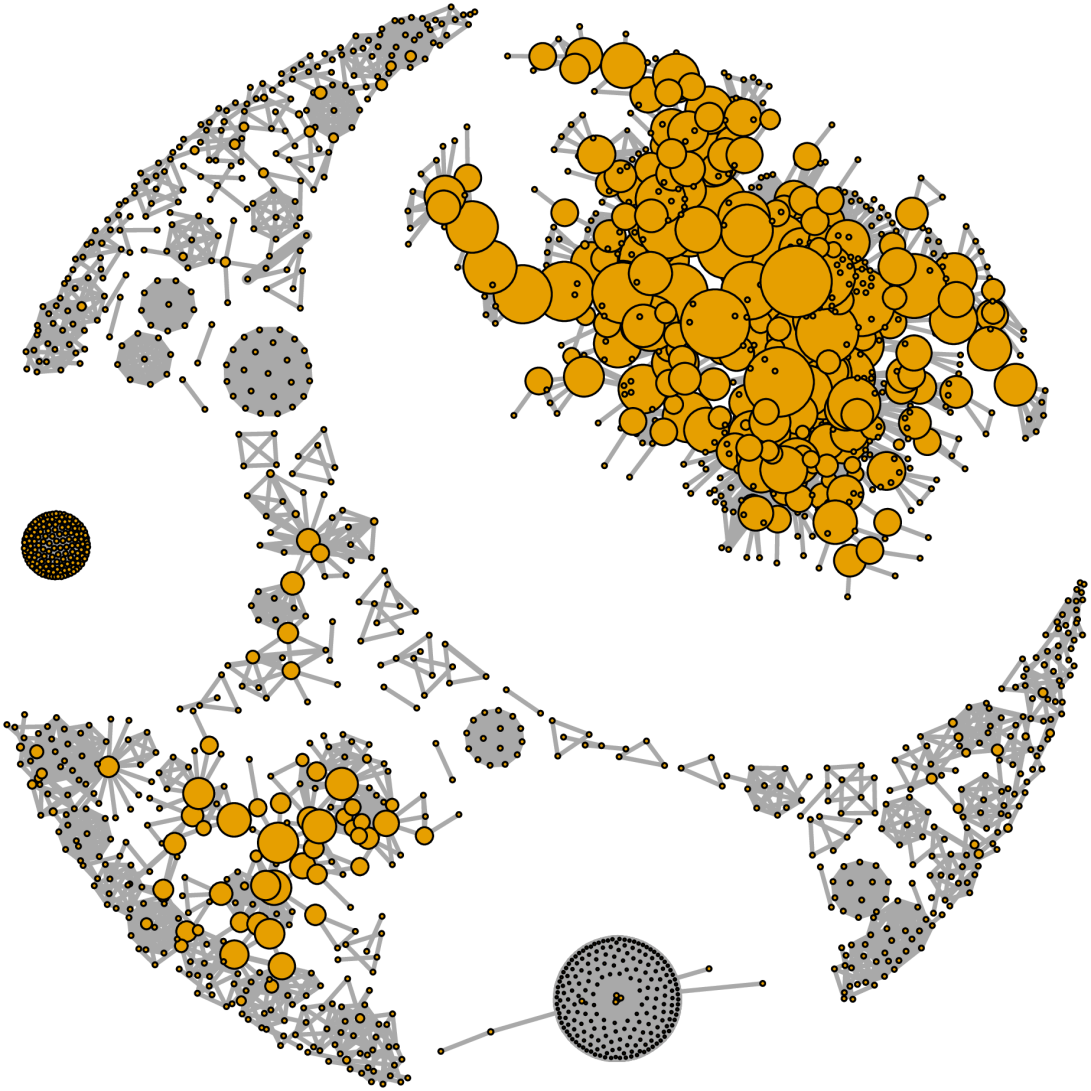
The citation network of peer review (Scopus data).

Fig 9 shows the co-citation network, which included 6402 articles and 71548 references. In the top-right hand corner, there is the community of 84 papers, while the two small clusters at the bottom-centre and middle-left, were examples of isolated co-citation links that were generated by a small number of articles (e.g., the bottom-centre was generated by four citing articles by the same authors with a high number of co-citation links). Table 3 presents the co-citation network metrics, including data on the giant component. Results suggest that the field is characterized by network fragmentation with 192 clusters with a limited size. While the giant component covered 33% of the nodes, it counted only 0.9% of the total number of cited sources in all records. Furthermore, data showed that 79.2% of co-citation links included no more than five cited references.

**Table 3:** Network statistics of the co-citation network and the giant component (Scopus data).

**Network metrics**	
**Number of nodes**	1829
**Number of edges**	36531
**Network density**	0.022
**Diameter**	33
**Number of clusters**	192
**Avg. Cluster size**	9.526
**Sd. Cluster size**	48.232
**Giant component**	
**Coverage (% nodes)**	33.62%
**Number of nodes**	615
**Number of edges**	1925
**Network density**	0.010
**Diameter**	33

Table 4 shows a selection of the most important references that were instrumental in clustering the co-citation network as part of the giant component. Results demonstrated not only the importance of certain classical sociology of science contributions, e.g., Robert Merton’s work, which showed an interest on peer review since the 1970s; also more recent works, including literature reviews, were important to decrease the disconnection of scientists in the field [2]. They also show that, at least for the larger co-citation subnetwork, the field is potentially inter-disciplinary, with important contributions from scholars in medicine as well as scholars from sociology and behavioural sciences.

**Table 4:** The most influential articles in the co-citation networks (Scopus data).

1	Merton, R.K. (1973), The Sociology of Science: Theoretical and Empirical Investigations, Chicago: University of Chicago Press
2	Zuckerman, H., Merton, R.K. (1971), Patterns of evaluation in science: Institutionalisation, structure and functions of the referee system. Minerva, 9, pp. 66–100
3	Horrobin, D.F. (1990), The philosophical basis of peer review and the suppression of innovation. Journal of the American Medical Association, 263, pp. 1438–1441
4	Bornmann, L. (2011), Scientific peer review. Annual Review of Information Science and Technology, 45, pp. 199–245
5	Siegelman, S.S. (1991), Assassins and zealots: Variations in peer review. Radiology, 178, pp. 637–642
6	Oppenheim, C. (1997), The correlation between citation counts and the 1992 research assessment exercise ratings for British research in genetics, anatomy and archaeology. Journal of Documentation, 53, pp. 477–487
7	Crane, D. (1967), The gatekeepers of science: Some factors affecting the selection of articles for scientific journals. American Sociologist, 32, pp. 195–201
8	Ingelfinger, F.J. (1974), Peer review in biomedical publication. Am J Med, 56, pp. 686–692
9	Peters, D.P., Ceci, S.J. (1982), Peer-review practices of psychological journals: The fate of published articles, submitted again. Behavioral and Brain Sciences, 5, pp. 187–255
10	Cole, S., Cole, J.R., Simon, G.A. (1981), Chance and consensus in peer review. Science, 214, pp. 881–886
11	Cronin, B., McKenzie, G. (1992), The trajectory of rejection. Journal of Documentation, 48 (3), pp. 310–317
12	Starbuck, W.H. 2003), Turning lemons into lemonade: Where is the value in peer reviews? Journal of Management Inquiry, 12, pp. 344–351
13	Wenneras, C., Wold, A. (1997), Nepotism and sexism in peer-review. Nature, 387, pp. 341–343
14	Lawrence, P.A. (2003), The politics of publication. Nature, 422, pp. 259–261
15	Travis, G.D.L., Collins, H.M. (1991), New light on old boys: Cognitive and institutional particularism in the peer review system. Science, Technology, & Human Values, 16 (3), pp. 322–341
16	Burnham, J.C. (1990), The evolution of editorial peer review. JAMA, 263, pp. 1323–1329
17	Van Raan, A.F.J. (2006), Comparison of the Hirsch-index with standard bibliometric indicators and with peer judgment for 147 chemistry research groups. Scientometrics, 67 (3), pp. 491–502
18	Aksnes, D.W., Taxt, R.E. (2004), Peer reviews and bibliometric indicators: A comparative study at a Norwegian university. Research Evaluation, 13 (1), pp. 33–41
19	Rothwell, P.M., Martyn, C.N. (2000), Reproducibility of peer review in clinical neuroscience: Is agreement between reviewers any greater than would be expected by chance alone? Brain, 123, pp. 1964–1969
20	Seng, L.B., Willett, P. (1995), The citedness of publications by United Kingdom library schools. Journal of Information Science, 21 (1), pp. 68–71

## Discussion and conclusions

Our analysis showed that research on peer review has been rapidly growing, especially from 2005. Not only the number of publications increased; it did also the number of citations and so the impact of research on peer review [18]. We also found that research is international, with more tradition in the US but with important research groups also in Europe. However, when looking at co-authorship networks, findings indicate that research is fragmented. Scholars do not collaborate on a significant scale, with the largest group of co-authors including only 2.1% of the whole community. When looking at co-citation patterns, we found that also knowledge sharing is fragmented. The larger networks covers only 33% of the nodes, which count only for 0.9% of the total number of cited sources in all records.

This calls for a serious consideration of certain structural problems of studies on peer review. First, difficulties in accessing data from journals and funding agencies and performing large-scale quantitative research have probably limited collaborative research [19]. While the lack of data may also be due to the confidentiality and anonymity that characterize peer review, it is also possible that editorial boards of journals and administrative bodies of funding agencies have interests in obstructing independent research as a means to protect internal decisions [8]. However, the full digitalisation of editorial management processes and the increasing emphasis on open data and research integrity among science stakeholders are creating a favourable context in which researchers will be capable of accessing peer review data more frequently and easily soon [20]. This is expected to stimulate collaboration and increase the scale of research on peer review. Secondly, the lack of specific funding schemes that support research on peer review has probably obstructed the possibility of systematic studies [10]. This has probably made difficult for scholars to establish large-scale, cross-disciplinary collaboration.

In conclusion, although peer review may reflect context-specificities and disciplinary traditions, the common challenge of understanding the complexity of this process, testing the efficacy of different models in reducing bias and allocating credit and reputation fairly requires ensuring comparison and encouraging collaboration and knowledge sharing across communities [21]. Here, a recently released document on data sharing by a European project has indicated that data sharing on peer review is instrumental to promote the quality of the process, with relevant collective benefits [22]. Not only such initiatives are important to improve the quality of research; they can also promote an evidence-based approach to peer review reorganizations and innovations, which is now not so well developed.

## Methods

Our sample included all records on peer review published from 1969 to 2015, which were extracted from Scopus on July 1^st^ 2016. We used the Advanced Search tab on the Scopus website to run our query strings (for detail, see below) and exported all available fields for each document retrieved as a CSV (comma separated values format) file. After several tests and checks on the dataset, we identified three samples of records that were hierarchically linked as follows:

1. Sample 1 (n = 6402 documents), which included any paper reporting “peer review” either in the “article title” or “author keywords” fields (the use of other fields, such as “Abstract” and “Keywords”, led to a high number of documents that reported about peer review but were excluded from the sample as we verified that they were not studies on peer review but just papers that had gone through a peer review process). This sample was obtained after deduplication of the following query to Scopus: (TITLE("peer review") OR AUTHKEY("peer review")) AND PUBYEAR < 2016.
2. Sample 2 (n = 3725 documents), which restricted sample 1 to journal articles (already published or just available online), books, book chapters and conference papers, so excluding editorial notes, reviews, commentaries and letters. This sample was obtained after deduplication of the following query to Scopus: (TITLE("peer review") OR AUTHKEY("peer review")) AND (DOCTYPE("ar") OR DOCTYPE ("ip") OR DOCTYPE ("bk") OR DOCTYPE ("ch") OR DOCTYPE ("cp")) AND PUBYEAR < 2016.
3. Sample 3 (n = 1955 documents), which restricted sample 2 to records that were not listed among “Medicine” as subject area. This sample was obtained after deduplication of the following query to Scopus: (TITLE("peer review") OR AUTHKEY("peer review")) AND (DOCTYPE("ar") OR DOCTYPE ("ip") OR DOCTYPE ("bk") OR DOCTYPE ("ch") OR DOCTYPE ("cp")) AND PUBYEAR < 2016 AND NOT(SUBJAREA("MEDI")).

With sample 1, we aimed to exclude records that were not explicitly addressed to peer review as an object of study. With sample 2, we identified only articles that reported results, data or cases. With sample 3, we aimed to understand specificities and differences between studies on peer review in medicine and other studies. If not differently mentioned, we reported results on sample 1. Note that, in order to check data consistency, we compared our Scopus dataset with other datasets and repositories, such as PubMed and WoS (see Figure A in S1 Appendix).

The queries to Scopus proposed in this paper allowed us to retrieve the corpus at a sufficient level of generality to look at the big picture of this field of research. Querying titles and author keywords about “peer review” did not restrict the search only to specific aspects, contexts or cases in which peer review could have been studied (e.g., peer review of scientific manuscripts). Although these queries could filter out some relevant papers, we strongly believe these cases had only a marginal impact on our analysis. For instance, we tried to use other related search terms and found a few papers from Scopus (e.g. just 2 documents for “grant decision making” and 3 documents for “grant selection”) and a number of false positives (e.g. the first 20 of the 69 documents obtained for “panel review” did not really deal with peer review as a field of research).

In order to visualize the collaboration structure in the field, we calculated co-authorship networks [23] in all samples. Each node in the co-authorship network represented a researcher, while each edge connected two researchers who co-authored a paper. In order to map knowledge sharing, we extracted co-citation networks [24–26]. In this case, nodes represented bibliographic references while edges connected two references when they were cited in the same paper. These methods are key to understand the emergence and evolution of research on peer review as a field driven by scientists’ connections and knowledge flows [27].

When constructing co-authorship and co-citation networks, we only used information about documents explicitly dealing with “peer review”. The rationale behind this decision was that we wanted to measure the kind of collaboration that can be attributed to these publications, regardless the total productivity of the scientists involved. Minor data inconsistencies can also happen due to the data exported from Scopus, WoS and PubMed not being complete, clean and free of errors. If a paper is missing, all co-authorship links that can be derived will be missing too. If an author name is written in two ways, two different nodes will represent the same researcher and links will be distributed between them. The continuous refinement, sophistication and precision of the algorithms behind these databases ensure that the amount of mistakes and missing information is irrelevant for a large-scale analysis. In any case, we implemented automatic mechanisms that cleaned data and removed duplicated records that reduced these inconsistencies to a marginal level given the scope of our study (see the R script used to perform the analysis in S1 File).

Research has extensively used co-authorship and co-citation networks to study collaboration patterns by means of different network descriptors [28]. Here, we focussed on the following indicators, which were used to extract information from the samples presented above:

- Number of nodes: the number of different co-authors and different bibliographic references, which was used to provide a structural picture of the community of researchers studying peer review.
- Number of edges: the sum of all different two-by-two relationships between researchers and between bibliographic references, which was used to represent the collaboration structure of this field of research.
- Network density: the ratio between the number of edges in the co-authorship network and the total number of edges that this network would have if it were completely connected, which was used to understand whether the community was cohesive or fragmented (i.e., higher the ratio, the more cohesive was the research community).
- Diameter: the longest shortest path in the network, which indicated the distance between its two farthest nodes (i.e., two authors or two references), so showing the degree of separation in the network.
- Betweeness centrality: the number of shortest paths between any two nodes that passed through any particular node of the network. Note that nodes around the edge of the network would typically have a lower betweeness centrality, whereas a higher betweeness centrality would indicate, in our case, that a scientist or a paper was connecting, respectively, different parts of the co-authorship or the co-citation network, thus playing a central role in connecting the community.
- Number and size of clusters: here, we found the number and size of densely connected sub-communities in the network after performing a simple breadth-first search. We used these indicators to understand if the community was characterized by a multitude of sub-groups relatively independent from each other, with someone sub-connecting more researchers.

## Supporting information

S1 Appendix (DOCX)

S1 File R code script used to perform the quantitative analysis. (R)
