# Supplementary material for: Fragments of peer review: A quantitative analysis of the literature (1969-2015)
Source: PLoS One. 2018 Feb 21;13(2):e0193148. doi: 10.1371/journal.pone.0193148 (PMC5821355; doi:10.1371/journal.pone.0193148)
Supplement: S1 Appendix — (DOCX) [file pone.0193148.s001.docx]

# S1 Appendix

In order to check the consistency of our results on Scopus, we performed the same analysis on other scientific databases and repositories, such as PubMed and Web of Science. PubMed uses MeSH (Medical Subject Headings) as a vocabulary thesaurus for article indexing, which includes “Peer Review, Research” as a category. By querying PubMed using the search string “Peer Review[MeSH Major Topic]” on its website and downloading outcomes as CSV, we obtained 7,121 documents from 1969 to 2015. Fig A corroborates our findings on the influence of the International Congress on Peer Review and Biomedical Publication. The same growing effect that we found in Scopus was also found in PubMed and WoS. A similar dynamics can be observed looking at the data from the Web of Science Core Collection (4,059 documents with titles including “peer review” are obtained by running the search string TITLE: (“peer review”) for the timespan 1900-2015 and saving the full record of each document in the results as CSV), although with less pronounced peaks due to the fact that the sample was not restricted only to medicine. Furthermore, WoS indexes a smaller number of publication outlets and only basic query facilities are available (e.g., search by author keywords or abstract are not present).


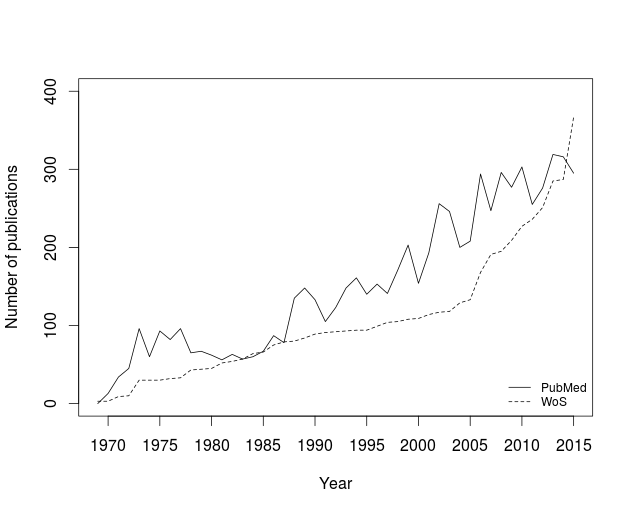


**Fig A. Papers on peer review indexed by PubMed and Web of Science.**

Fig B shows the type of records that were published over time. About half of the records were journal articles, the rest mostly being editorial notes, commentaries, letters and literature reviews. According to the classification provided by subject areas in Scopus, 49.84% of records were published in Medical journals, with an additional 8.02% in Nursing, 20.86% in Social Science journals and 8.27% in Computer Science journals. Only 4.8% of papers were published in multi-disciplinary journals and 31.19% were published in journals from 17 other disciplines such as Environmental Sciences, Mathematics, Physics, Economics and Chemistry (note that Scopus can assign different subject areas to the same journal).


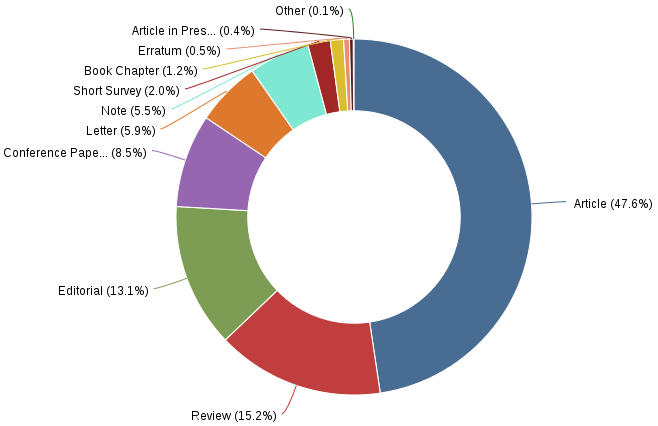


**Fig B. Percentage of type of records on the total number of documents (Scopus data).**

Table A shows the top journals for number of citations collected by articles on peer review, in which the prominent case of *Scientometrics* is evident. Table B provides a more dynamic picture in that for almost all journals listed in the top 10, publications on peer review received higher citations over time compared to citations received by journal articles.

**Table A. Top journals for number of citations collected by papers on peer review.**

| **Journal name** | **Citations of papers on peer review** | **Total journal citations extracted from JCR** | **% of citations of papers on peer review on the total number of journal citations** |
| --- | --- | --- | --- |
| *Journal of the American medical association (JAMA)* | 3134 | 129909 | 2.41 |
| *Journal of Clinical Epidemiology* | 934 | 20734 | 4.50 |
| *British Medical Journal (BMJ)* | 809 | 93118 | 0.87 |
| *Nature* | 741 | 627846 | 0.12 |
| *Scientometrics* | 706 | 6436 | 10.97 |
| *Science* | 706 | 568210 | 0.12 |
| *Medical Education* | 657 | 7539 | 8.34 |
| *Behavioral and Brain Sciences* | 540 | 7873 | 6.86 |
| *Research Policy* | 438 | 13078 | 3.35 |
| *Medical Care* | 431 | 16813 | 2.56 |

In order to calculate the number of citations of sample 1 papers, we used the field Citation count in Scopus.

Table C shows the most cited papers on peer review in Scopus. These include research articles with empirical or experimental research, e.g., “Validation of an index of the quality of review articles” by Oxmad and Guyatt, the most cited paper and “Nepotism and sexism in peer-review” by Wennerås and Wold. This indicates that the most influential papers were research articles rather than editorial notes, commentaries or literature reviews. It is important to note that we did not find any significant differences of ranking of the most cited records when controlling for time effects, i.e., weighting citations per number of years from publication.

**Table B. Annual evolution of the ratio of citations of papers on peer review on the total number of journal citations.**

|  | **<2005** | **2005** | **2006** | **2007** | **2008** | **2009** | **2010** | **2011** | **2012** | **2013** | **2014** | **2015** |
| --- | --- | --- | --- | --- | --- | --- | --- | --- | --- | --- | --- | --- |
| **JAMA** | 1,34% | 1,38% | 1,51% | 1,64% | 1,63% | 1,75% | 1,91% | 2,05% | 2,12% | 2,23% | 2,33% | 2,41% |
| **Epidemiology** | 2,74% | 2,67% | 2,90% | 3,21% | 3,26% | 3,77% | 3,94% | 4,15% | 4,25% | 4,12% | 4,29% | 4,50% |
| **BMJ** | 0,40% | 0,43% | 0,52% | 0,62% | 0,64% | 0,69% | 0,76% | 0,81% | 0,82% | 0,84% | 0,86% | 0,87% |
| **Nature** | 0,05% | 0,05% | 0,06% | 0,06% | 0,07% | 0,07% | 0,08% | 0,09% | 0,09% | 0,10% | 0,11% | 0,12% |
| **Scientometrics** | 8,95% | 6,69% | 8,32% | 8,91% | 7,14% | 5,96% | 6,91% | 7,46% | 8,10% | 9,34% | 10,03% | 10,97% |
| **Science** | 0,07% | 0,07% | 0,08% | 0,08% | 0,08% | 0,08% | 0,08% | 0,08% | 0,08% | 0,09% | 0,10% | 0,12% |
| **Medical Education** | 2,76% | 3,61% | 4,94% | 5,55% | 6,54% | 6,65% | 7,05% | 8,07% | 8,43% | 8,06% | 9,62% | 8,71% |
| **Beh Brain Sciences** | 5,27% | 5,81% | 5,81% | 5,87% | 5,84% | 5,52% | 6,37% | 6,74% | 7,00% | 6,87% | 6,65% | 6,86% |
| **Research Policy** | 2,09% | 2,31% | 2,65% | 2,61% | 2,17% | 2,22% | 2,51% | 3,02% | 2,97% | 3,43% | 3,44% | 3,35% |
| **Medical Care** | 2,77% | 2,73% | 2,51% | 2,69% | 2,58% | 2,58% | 2,65% | 2,59% | 2,53% | 2,55% | 2,51% | 2,56% |

In order to calculate the number of citations of sample 1 papers, we used the field Citation count in Scopus.

**Table C. The top cited articles on peer review**.

| **Title** | **Authors** | **Journal** | **Publication year** | **Citations** |
| --- | --- | --- | --- | --- |
| Validation of an index of the quality of review articles | Oxmad, A.D. and Guyatt, G.H. | Journal of Clinical Epidemiology | 1991 | 464 |
| Nepotism and sexism in peer-review | Wennerås, C. and Wold, A. | Nature | 1997 | 432 |
| Publication prejudices: An experimental study of confirmatory bias in the peer review system | Mahoney, M.J. | Cognitive Therapy and Research | 1977 | 377 |
| Peer-review practices of psychological journals: The fate of published articles, submitted again | Peters, D.P. and Ceci, S.J. | Behavioral and Brain Sciences | 1982 | 326 |
| Who is afraid of peer review? | Bohannon, J. | Science | 2013 | 231 |
| The philosophical basis of peer review and the suppression of innovation | Horrobin, D.F. | JAMA | 1990 | 219 |
| The power of gifts: Organizing social relationships in open source communities | Bergquist, M. and Ljungberg, J | Information Systems Journal | 2001 | 210 |
| The reliability of peer review for manuscript and grant submissions: A cross-disciplinary investigation | Cicchetti, D.V. | Behavioral and Brain Sciences | 1991 | 210 |
| Chance and consensus in peer review | Cole, S., Cole, J.R. and Simon, G.A. | Science | 1981 | 207 |
| Advanced bibliometric methods as quantitative core of peer review based evaluation and foresight exercises | Van Raan, A.F.J. | Scientometrics | 1996 | 187 |

In order to calculate the number of citations of sample 1 papers, we used the field Citation count in Scopus.

Fig C compares data on the most prolific authors in sample 2 (only research articles) and sample 3 (outside medicine), to complement Fig 5 included in the main text. Although there are some differences, the top two most prolific authors were still the same. While the number of publications per author and year ranged from 0 to12, with most authors publishing 1 to 4 articles, the two most prolific authors published 12 and 9 articles respectively, in 2010.


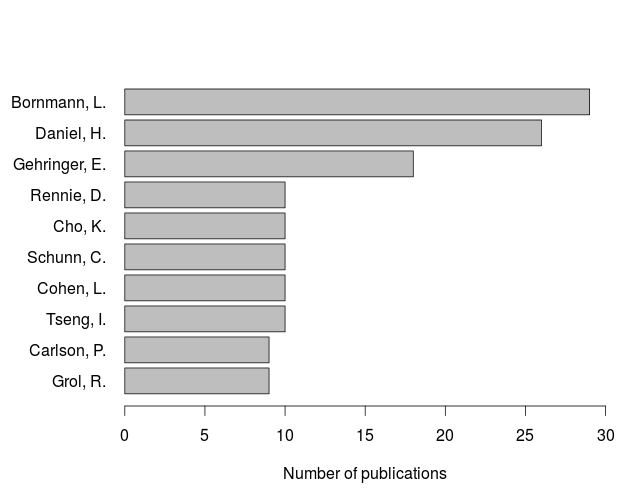

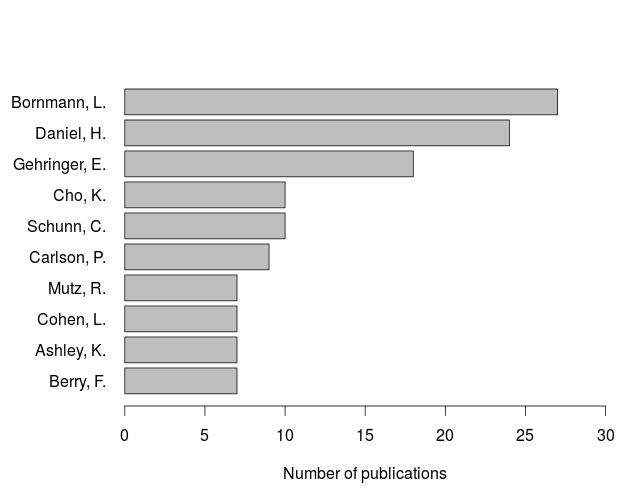


**Fig C. The top 10 most prolific authors.** On the left, the most prolific in sample 2 (only articles and conference papers). On the right, the most prolific in sample 3 (without medicine) (Scopus data).

Table D shows data on citations in the three samples, while Table E shows data on co-authorship (column Diff refers to the number of different co-authors). It is interesting to note that the most prolific authors were also those with more co-authors, though they were not those with a higher average of co-authors per paper. Please, note that the number of co-authors is influenced by two cases (i.e., two articles with 32 and 26 co-authors respectively) that benefit scientists of single papers with many co-authors at the expense of hiding other more social authors. Table F calculates the same indicators shown in Table E but after removing any record with more than 10 co-authors, as records higher than these thresholds predominate and are not representative, e.g., 55 cases with more than 10 co-authors against 6,347 cases with less.

**Table D. The top 10 most cited scientists in the three samples (Scopus data).**

| **Sample 1** | |  | **Sample 2** | |  | **Sample 3** | |
| --- | --- | --- | --- | --- | --- | --- | --- |
| **Author** | **Citations** |  | **Author** | **Citations** |  | **Author** | **Citations** |
| Smith, Richard | 884 |  | Oxman, Andrew D. | 633 |  | Bornmann, Lutz | 562 |
| Bornmann, Lutz | 818 |  | Guyatt, Gordon H. | 633 |  | Ceci, Stephen J. | 497 |
| Godlee, Fiona | 711 |  | Godlee, Fiona | 603 |  | Daniel, Hans D. | 406 |
| Oxman, Andrew D. | 633 |  | Bornmann, Lutz | 580 |  | Mahoney, Michael J. | 380 |
| Guyatt, Gordon H. | 633 |  | Ceci, Stephen J. | 520 |  | Van Raan, Anthony F.J. | 355 |
| Daniel, Hans D. | 621 |  | Van Rooyen, Susan | 470 |  | Peters, Douglas P. | 325 |
| Black, Nicholas A. | 524 |  | Daniel, Hans D. | 425 |  | Schunn, Christian D. | 261 |
| Ceci, Stephen J. | 520 |  | Black, Nicholas A. | 424 |  | Cole, Stephen | 247 |
| Evans, Stephen J. W. | 501 |  | Smith, Richard | 412 |  | Cole, Jonathan R. | 247 |
| Van Rooyen, Susan | 486 |  | Evans, Stephen J. W. | 401 |  | Cho, Kwangsu | 242 |

**Table E. The top 10 scientists for number of co-authors in the three samples (Scopus data).**

| **Sample 1** | | | |  | **Sample 2** | | | |  | **Sample 3** | | | |
| --- | --- | --- | --- | --- | --- | --- | --- | --- | --- | --- | --- | --- | --- |
| **Author** | **Total** | **Diff** | **Avg** |  | **Author** | **Total** | **Diff** | **Avg** |  | **Author** | **Total** | **Diff** | **Avg** |
| Braun, J.P. | 71 | 36 | 6.3 |  | Johnson, N. | 51 | 37 | 17 |  | Bornmann,L. | 40 | 11 | 1.5 |
| Bornmann,L. | 64 | 21 | 1.5 |  | Braun, J.P. | 42 | 27 | 7.7 |  | Bollen, J. | 36 | 34 | 9 |
| Bause, H. | 64 | 33 | 9.1 |  | Bornmann,L. | 42 | 11 | 1.5 |  | Daniel, H.D. | 36 | 8 | 1.5 |
| Bloos, F. | 59 | 30 | 14.8 |  | Brinkmann, A. | 40 | 25 | 13.3 |  | Kriegler, E. | 31 | 31 | 31 |
| Waydhas, C. | 59 | 30 | 14.8 |  | Dubb, R. | 40 | 25 | 13.3 |  | Riahi, K. | 31 | 31 | 31 |
| Spies, C. | 59 | 30 | 14.8 |  | Kaltwasser, A. | 40 | 25 | 13.3 |  | Bauer, N. | 31 | 31 | 31 |
| Daniel, H.D. | 53 | 12 | 1.5 |  | Daniel, H.D. | 38 | 8 | 1.5 |  | Schwanitz, V.J. | 31 | 31 | 31 |
| Johnson, N. | 51 | 41 | 17 |  | Bollen, J. | 36 | 34 | 9 |  | Petermann, N. | 31 | 31 | 31 |
| Bollen, J. | 44 | 38 | 7.3 |  | Bause, H. | 36 | 23 | 12 |  | Bosetti, V. | 31 | 31 | 31 |
| Rennie, D. | 42 | 13 | 1.8 |  | Chop, I. | 36 | 23 | 9 |  | Marcucci, A. | 31 | 31 | 31 |

**Table F. The top 10 scientists for number of co-authors in the three samples (Scopus data) restricted to papers with up to 10 co-authors.**

| **Sample 1**  **[nº Authors <=10]** | | | |  | **Sample 2**  **[nº Authors <=10]** | | | |  | **Sample 3**  **[nº Authors <=10]** | | | |
| --- | --- | --- | --- | --- | --- | --- | --- | --- | --- | --- | --- | --- | --- |
| **Author** | **Total** | **Diff** | **Avg** |  | **Author** | **Total** | **Diff** | **Avg** |  | **Author** | **Total** | **Diff** | **Avg** |
| Bornmann,L. | 64 | 21 | 1.5 |  | Bornmann,L. | 42 | 11 | 1.5 |  | Bornmann,L. | 40 | 11 | 1.5 |
| Daniel, H.D. | 53 | 12 | 1.6 |  | Daniel, H.D. | 38 | 8 | 1.5 |  | Daniel, H.D. | 36 | 8 | 1.5 |
| Rennie, D. | 42 | 13 | 1.8 |  | McKay, J.S. | 30 | 21 | 4.3 |  | Gehringer, E. | 23 | 19 | 1.3 |
| McKay, J.S. | 41 | 26 | 4.6 |  | Bowie, P. | 27 | 16 | 3.4 |  | Carlson, P. | 21 | 17 | 2.3 |
| Flanagin, A. | 39 | 15 | 2.6 |  | Gehringer, E. | 23 | 19 | 1.3 |  | Schunn, C.D. | 18 | 14 | 1.8 |
| Godlee, F. | 36 | 13 | 3.3 |  | Marusic, A. | 22 | 17 | 4.4 |  | Hamasaki, K. | 17 | 8 | 5.7 |
| Bowie, P. | 35 | 22 | 3.2 |  | Grol, R. | 22 | 17 | 2.4 |  | Kula, R.G. | 17 | 8 | 5.7 |
| Grol, R. | 35 | 28 | 2.7 |  | Carlson, P. | 21 | 17 | 2.3 |  | Yoshida, N. | 17 | 8 | 5.7 |
| Marusic, A. | 32 | 22 | 3.6 |  | Lough, M. | 21 | 11 | 3.5 |  | Fujiwara, K. | 17 | 8 | 5.7 |
| Perry, G. | 32 | 8 | 8 |  | Godlee, F. | 19 | 9 | 3.2 |  | Iida, H. | 17 | 8 | 5.7 |

Fig D confirms that research on peer review is typically pursued in small collaborative networks. In sample 1, it is possible to note the exception of an article published in 2012, which was co-authored by 198 authors. Fig E shows that in sample 1 82.7% of co-authorship sub-networks had no more than five authors, 82.8% in sample 2, 88.9% in sample 3, confirming that research collaboration on studying peer review has been mainly small-scale.


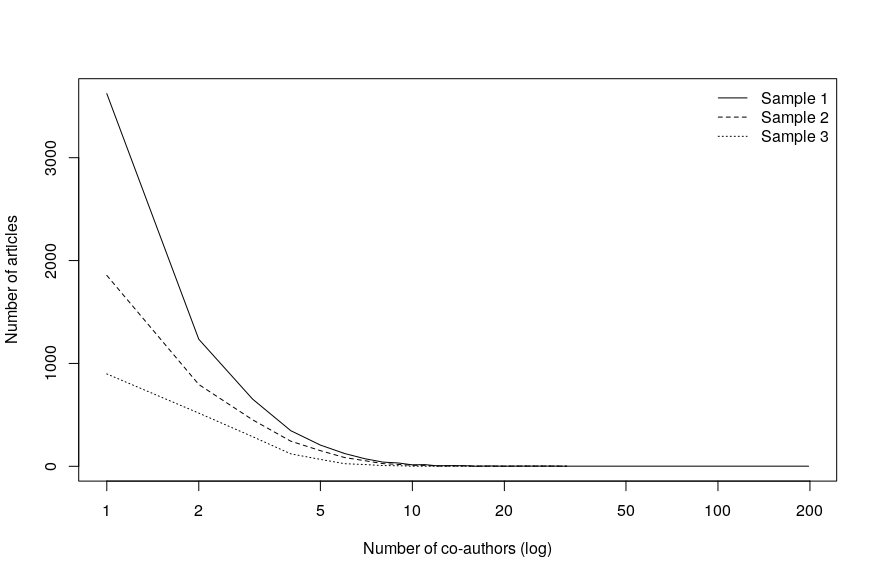


**Fig D. Ratio between the number of articles and the number of co-authors in all samples.** (Scopus data).


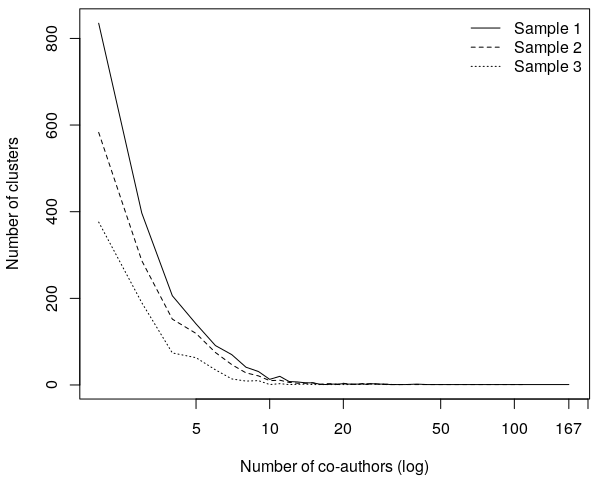


**Fig E. The size distribution of co-authorship networks of scientists working on peer review.** (Scopus data).

Finally, Fig F shows that most co-citation networks included less than five articles, confirming the lack of robust and generalized knowledge sharing patterns among scientists doing research on peer review.


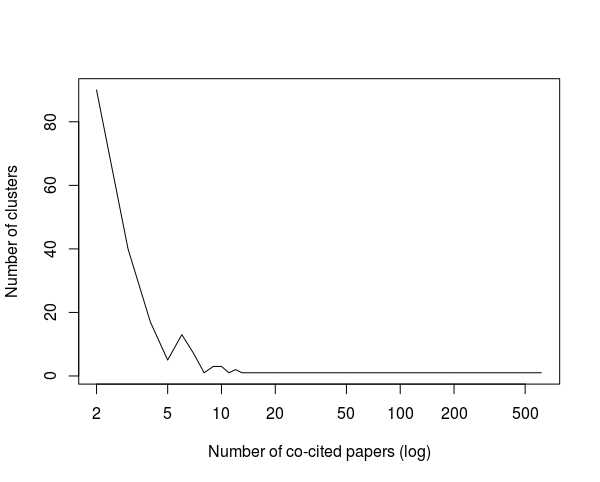


**Fig F. Co-citation clusters** (Scopus data).
